# Spontaneous vertebral artery dissection with multiple supratentorial and infratentorial acute infarcts in the posterior circulation Case report

**Published:** 2016

**Authors:** I Cristea, C Popa

**Affiliations:** * Neurologic Clinic, National Institute of Neurology and Cerebrovascular Diseases, Bucharest, Romania; “Carol Davila” University of Medicine and Pharmacy, Bucharest, Romania; ** ”Carol Davila” University of Medicine and Pharmacy, Bucharest, Romania; Romanian Society of Stroke; Neurology Department, National Institute of Neurology and Cerebrovascular Diseases, Bucharest, Romania

**Keywords:** vertebral artery dissection, lateral medullar infarction, posterior inferior cerebellar artery

## Abstract

The article represents a case of a young patient with atypical clinical and paraclinical presentation of vertebral artery dissection by multiple cerebral infarcts, localized at the supratentorial and infratentorial levels in the posterior circulation.

A case of a 21-year-old man, without a history of trauma in the cervical area or at the cranial level, without recent chiropractic maneuvers or practicing a sport, which required rapid, extreme, rotational movements of the neck, was examined. He presented to the emergency room with nausea, numbness of the left limbs, dysarthria, and incoordination of walking, with multiple objective signs at the neurological examination, which revealed right vertebral artery subacute dissection after the paraclinical investigations. The case was particular due to the atypical debut symptomatology, through the installation of the clinical picture in stages, during 4 hours and by multiple infarcts through the artery-to-artery embolic mechanism in the posterior cerebral territory.

**Abbreviations**:

PICA = posterior inferior cerebellar artery, CT = computed tomography, MRI = magnetic resonance imaging, angio MRI = mangnetic resonance angiography, FLAIR = fluid attenuated inversion recovery, FS = fat suppression, ADC = apparent diffusion coefficient, DWI = diffusion weighted imaging, T1/ T2 = T1/ T2 weighted image-basic pulse sequences in MRI, VA = vertebral artery, 3D-TOF = 3D Time of Flight

## History

The 21-year-old patient presented to the closest emergency department at 8 PM for nausea, numbness of the left limbs, incoordination of walking and dysarthria, with the symptomatology installed in a progressive mode 4 hours before presentation. The nausea was the initial symptom, starting at 4 PM (without headache, nuchal pain, or vomit) and then, after 5.30 PM, the numbness of the left limbs and the incoordination of walking and by 7 PM dysarthria was added to the entire clinical constellation.

The patient did not have a medical history or a current medication. He was a student. He did not smoke and did not use recreational drugs. He only drank alcohol occasionally. There was no family history of thromboembolism, cardiovascular problems, or hematologic diseases.

## Clinical and paraclinical examination

He was first evaluated by a neurosurgeon in the emergency unit and had a brain CT scan that highlighted infracentimetric hypodensity in the thalamic nucleus, on the left side and at the level of the right cerebellar hemisphere in the PICA territory, and also a chest X-ray that revealed a widened projection area of the right side hilum, without any other pathological findings. Following this evaluation, he was sent to the National Institute of Neurology and Cerebrovascular Diseases for consultation and special treatment.

During the ambulance transportation, he had repeated episodes of vomiting.

The initial physical examination (at 10 P.M) showed normal vital signs: blood pressure 130/ 80mmHg, heart rate 100 beats per minute and normal body temperature 36,8°C. The cardiopulmonary exam was normal. No carotid bruits were registered. 

The neurological examination was notable for normal orientation. The patient had equal, reactive pupils, divergent strabismus to the left eye, horizontal nystagmus, normal deglutition, mild paresis of the left arm, ataxia of all limbs, with an accent on the left extremities, numbness of the left limbs, bilateral response present at all deep tendons reflex, Babinski sign on the left side, stereotypical language. The patient was able to repeat a few words and fragment of phrases and to perform simple commands of medium complexity.

The laboratory analysis highlighted a normal hemoleucogram, normal blood glucose level 9,11 mmol/ L (normal range 3,9-5,8mmol/ L), serum creatinine 0,69mmol/ L (range 0,5-0,9mmol/ L), total serum cholesterol 5,2mmol/ L (normal range < 5,2 mmol/ L). The tests for thrombophilia were negative. He also presented a normal sinus rhythm on the ECG. The brain CT performed at the first emergency unit, as mentioned above, revealed a infracentimetric hypodensity at the level of the left thalamic nucleus and at the level of the right cerebellar hemisphere in the PICA territory.

Doppler ultrasound for cerebral and cervical blood vessels highlighted the marked frena in the flow velocities of the V2 segment of the right vertebral artery, an accentuated aspect in the high V2 segments with the complete disappearance of the flow in the V3 segment, without carotid atheromatous lesions or modifications of the trajectories or the arterial caliber.

Overall, there was a possibility of a dissection of the right vertebral artery in the higher segments with indication of cerebral and angio MRI.

Cerebral and angio MRI showed: areas with T2, FLAIR hypersignal, with an important restriction of diffusion and low ADC, situated in the right cerebellar area of the postero-inferior territory, anterior bilateral cerebellar territory, left cerebellar peduncle, both right > left thalamic nuclei, as well as subcortical occipital bilateral, with an ischemic aspect that was recently constituted in the vertebrobasilar territory. T1 FS hypersignal was present on the entire intracranial segment of the right vertebral artery and the cranio-spinal junction. On the angiographic arterial 3D TOF sequence, the presence of the rapid flow of the right vertebral artery could be viewed, up to the C2 segment, where there was a progressive pinch out with an aspect of flute beak shape. Distal of this level, there was an absence of the rapid flow signal and also a lack of right PICA visualization. The MRI aspect suggested a subacute dissection of the right vertebral artery. The cerebral ventricular system had normal dimensions, shape, and topography. The pericerebral fluid spaces and the basal cisterns were normal. Conclusions: there were some ischemic lesions recently constituted in the vertebrobasilar territory and also a subacute dissection of the right vertebral artery. 

**Fig. 1:**
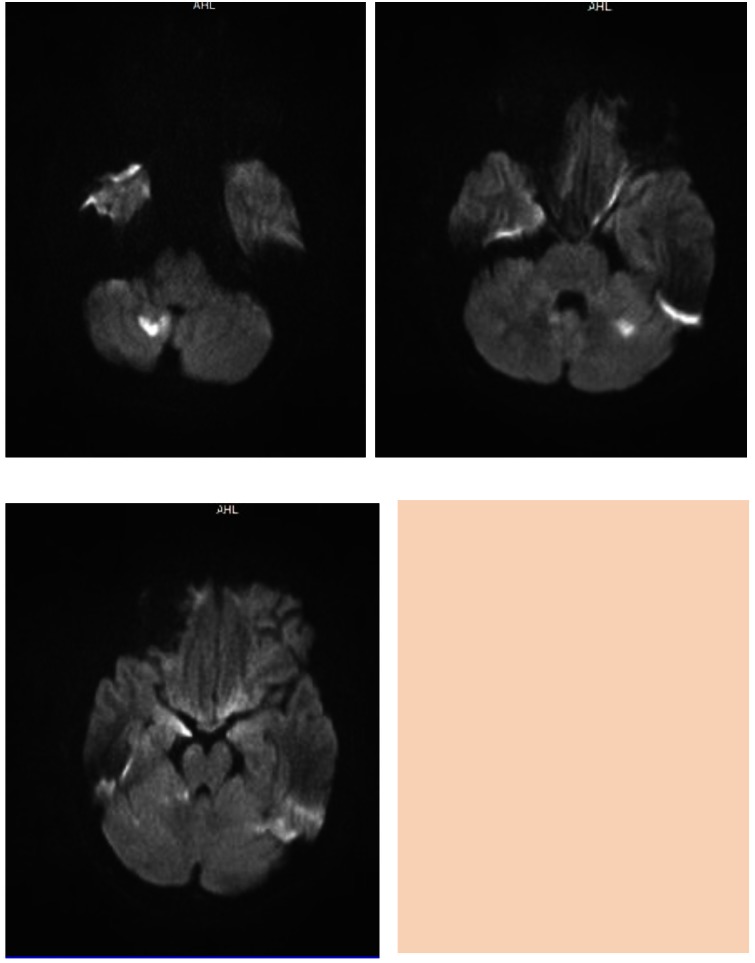
Acute Ischemic stroke in the cerebellar bilateral territory

**Fig. 2:**
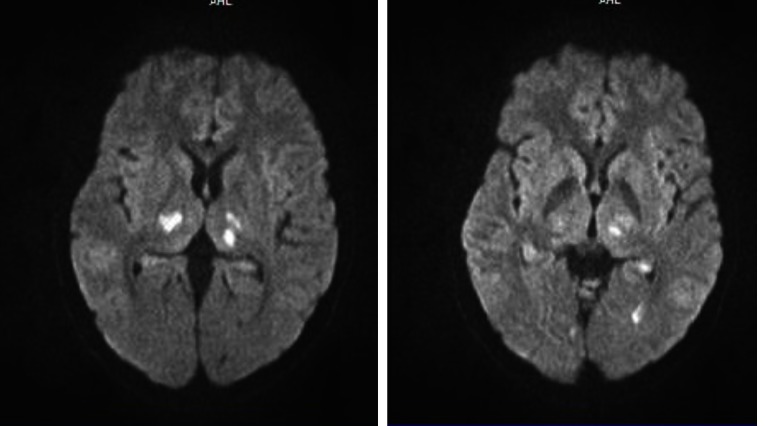
Acute ischemic stroke in both right and left thalamic nuclei, as well as in subcortical occipital bilateral territory

**Fig. 3:**
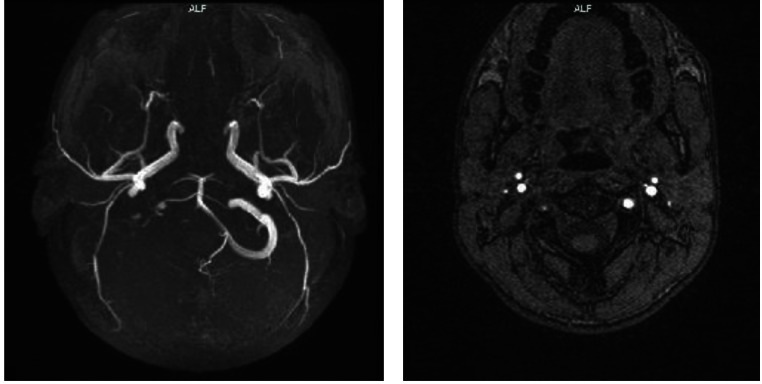
Subacute dissection of the right vertebral artery on the angiographic arterial 3D TOF sequence

Heparin administration was started immediately in the process of hospitalization because of the dissection. The APTT value was kept in the range of 50-70 seconds. Also, the prophylactic therapy for stress ulcer was performed. After 7 days from the debut, the patient was transitioned to acenocumarol for long-term anticoagulation. The treatment with acenocumarol was instituted with the heparin overlap until getting the INR therapeutic in the 2-3 intervals.

The evolution was favorable, the patient regaining the verbal fluency and the capacity for autonomy in movement. The ataxia was remitted at the right limbs and persisted at the left extremities, but registered a lot of improvement. The patient was discharged home with the recommendation to continue the treatment with Acenocumarol for 6 months with clinical, paraclinical and treatment reevaluation at 6 months.

The follow up was not possible because the patient did not return to the hospital after 6 months.

## Discussion

There was no precipitating factor of vertebral artery dissection in the case presented above, therefore it was considered a spontaneous vertebral artery dissection [**1**].

After the studies performed on multiples infarcts on the vertebrobasilar territory [**2**,**3**] it was observed that their etiology is generated most of the times by the involvement of the artery-to-artery embolism at the level of the vertebral artery. The dissection of the vertebral artery associated with multiple infarcts in the posterior territory had a bad prognosis as compared to the case of the classical clinical presentation with only lateral medullary infarct [**2**,**3**].

This category can also enclose the case described above, which presented an atypical debut of vertebral artery dissection with multiple infarcts in the posterior territory [**4**,**5**] by artery-to-artery embolism, but with a good evolution at two weeks from the debut, when the patient was discharged home.

## Conclusion

The presented case showed the importance of the knowledge of the posterior cerebral circulation, the clinical-paraclinical particularities of vertebral artery dissection and integrated the Wallenberg syndrome in a complex semiologic clinical picture.

**Acknowledgments**

Thanks and respect to Academician Professor Popa Constantin, MD, for the permanent and constant guidance. Without his involvement, this article would not have been possible. 

**Disclosures**

None
